# Widespread Environmental Presence of Multidrug-Resistant *Salmonella* in an Equine Veterinary Hospital That Received Local and International Horses

**DOI:** 10.3389/fvets.2020.00346

**Published:** 2020-07-10

**Authors:** Paula Soza-Ossandón, Dácil Rivera, Rodolfo Tardone, Roberto Riquelme-Neira, Patricia García, Christopher Hamilton-West, Aiko D. Adell, Gerardo González-Rocha, Andrea I. Moreno-Switt

**Affiliations:** ^1^Escuela de Medicina Veterinaria, Facultad de Ciencias de la Vida, Universidad Andres Bello, Santiago, Chile; ^2^Millennium Initiative for Collaborative Research on Bacterial Resistance (MICROB-R), Santiago, Chile; ^3^Facultad de Medicina, Pontificia Universidad Católica de Chile, Santiago, Chile; ^4^Unidad de Epidemiología Veterinaria, Departamento Medicina Preventiva Animal, Facultad de Ciencias Veterinarias y Pecuarias, Universidad de Chile, Santiago, Chile; ^5^Laboratorio de Investigación de Agentes Antimicrobianos, Departamento de Microbiología, Facultad de Ciencias Biológicas, Universidad de Concepción, Concepción, Chile; ^6^Escuela de Medicina Veterinaria, Pontificia Universidad Católica de Chile, Santiago, Chile

**Keywords:** *Salmonella enterica*, multidrug-resistant, equine hospital, hospital-acquired infections, biosecurity

## Abstract

*Salmonella enterica* is a highly infectious microorganism responsible for many outbreaks reported in equine hospitals. Outbreaks are characterized by high morbidity and mortality rates, nosocomial transmission to other patients, zoonotic transmission to hospital personnel, and even closure of facilities. In this study, 545 samples (environmental and hospitalized patients) were collected monthly during a 1-year period from human and animal contact surfaces in an equine hospital that received local and international horses. A total of 22 *Salmonella* isolates were obtained from human contact surfaces (e.g., offices and pharmacy) and animal contact surfaces (e.g., stalls, surgery room, and waterers), and one isolate from a horse. Molecular serotyping revealed 18 isolates as *Salmonella* Typhimurium and three as *Salmonella* Infantis. Nineteen isolates were resistant to at least one antimicrobial class, and only two isolates were susceptible to all antimicrobials tested. In addition, we identified nine multidrug-resistant (MDR) isolates in *S*. Typhimurium, which displayed resistance to up to eight antimicrobials (i.e., amoxicillin/clavulanate, ampicillin, ciprofloxacin, chloramphenicol, streptomycin, gentamicin, trimethoprim/sulfamethoxazole, and tetracycline). Pulsed-field gel electrophoresis (PFGE) revealed the presence of three PFGE patterns permanently present in the environment of the hospital during our study. The persistent environmental presence of MDR *Salmonella* isolates, along with the fact that local and international horses are attended in this hospital, highlights the importance of improving biosecurity programs to prevent disease in horses and the hospital personnel and also for the global dissemination and acquisition of MDR *Salmonella*.

## Introduction

*Salmonella enterica*, a Gram-negative bacteria of the family Enterobacteriaceae, is an important zoonotic pathogen that causes an estimated of 93.8 human cases and 150,000 deaths every year worldwide (1). *Salmonella* is usually transmitted to humans as foodborne and through contact with infected animals (2). This pathogen is a microorganism responsible for gastrointestinal disease affecting equines (among other animals) of all ages (3). Clinical symptoms include diarrhea, fever, and dehydration, with severity ranging from a subclinical colonization to a severe systemic illness (4). As a highly contagious disease, it can be reported as sporadic cases or as an outbreak (5, 6). Previous studies have reported significant mortality (38–44%) (7, 8) associated with salmonellosis outbreaks in equine veterinary hospitals (EVHs). Also, hospitalization and associated use of health-care resources increase the susceptibility of horses to strains of *S. enterica* disseminated by asymptomatic animals (4, 5).

It has been reported that one of the main reasons for the increasing rate of salmonellosis outbreaks are multidrug-resistant (MDR) strains of *Salmonella* (9–13). Last year, the New York State Veterinary Diagnostic Laboratory reported the isolation of *Salmonella* Group C2 from four different horse farms, which had shown the same MDR profile (14). This is rather concerning if we consider that back in the early 2000s, a strain of an MDR–*Salmonella* Newport (G2) was responsible of a serious outbreak in a Large Animal Teaching Hospital (9, 15). It is still unclear when or how MDR–*Salmonella* emerged, being one of the main suspects in the non-therapeutic use of antibiotics (14).

Salmonellosis outbreaks in animal health facilities are full of challenges beside the sole medical treatment and control the outbreak *per se*; they also involve communication with owners and referring veterinarians of infected horses (10). On the other hand, the consequences are serious including hospital-acquired infections of patients and hospital personnel, the establishment of expensive infection control programs, and decrease in clients' trust and hospitals' revenues and may even lead to litigation procedures (11, 12). Infection control programs should be an integral part of every animal health facility (16, 17). Several studies have reported outbreak control measurements (7, 12, 18) and assessment of protocols of contamination, which have been adopted by many facilities (16, 19). To date, there are no reports of salmonellosis in veterinary hospitals in Chile, and therefore, scarce biosecurity protocols have been established. Hence, this study was performed to determine the presence, antimicrobial resistance, and subtypes of *Salmonella* in the environment and patients from an EVH without reported history of outbreaks or hospital-acquired infections.

## Materials and Methods

### Description of the Setting and Location

The EVH is located at a thoroughbred horse racetrack at the center of the city of Santiago (Chile). It has an average flow of 100 incoming patients daily, providing equine health services to Thoroughbred, Arabian, Chilean rodeo, and Warmblood horses. This veterinary hospital has no records of outbreak or hospital-acquired infections due to *Salmonella* spp., and this information is remarkable in view of the lack of biosecurity measures or infection control programs (e.g., isolation of infected patients and protocols for cleaning and sanitation).

### Sampling Procedure

**Figure 1:**
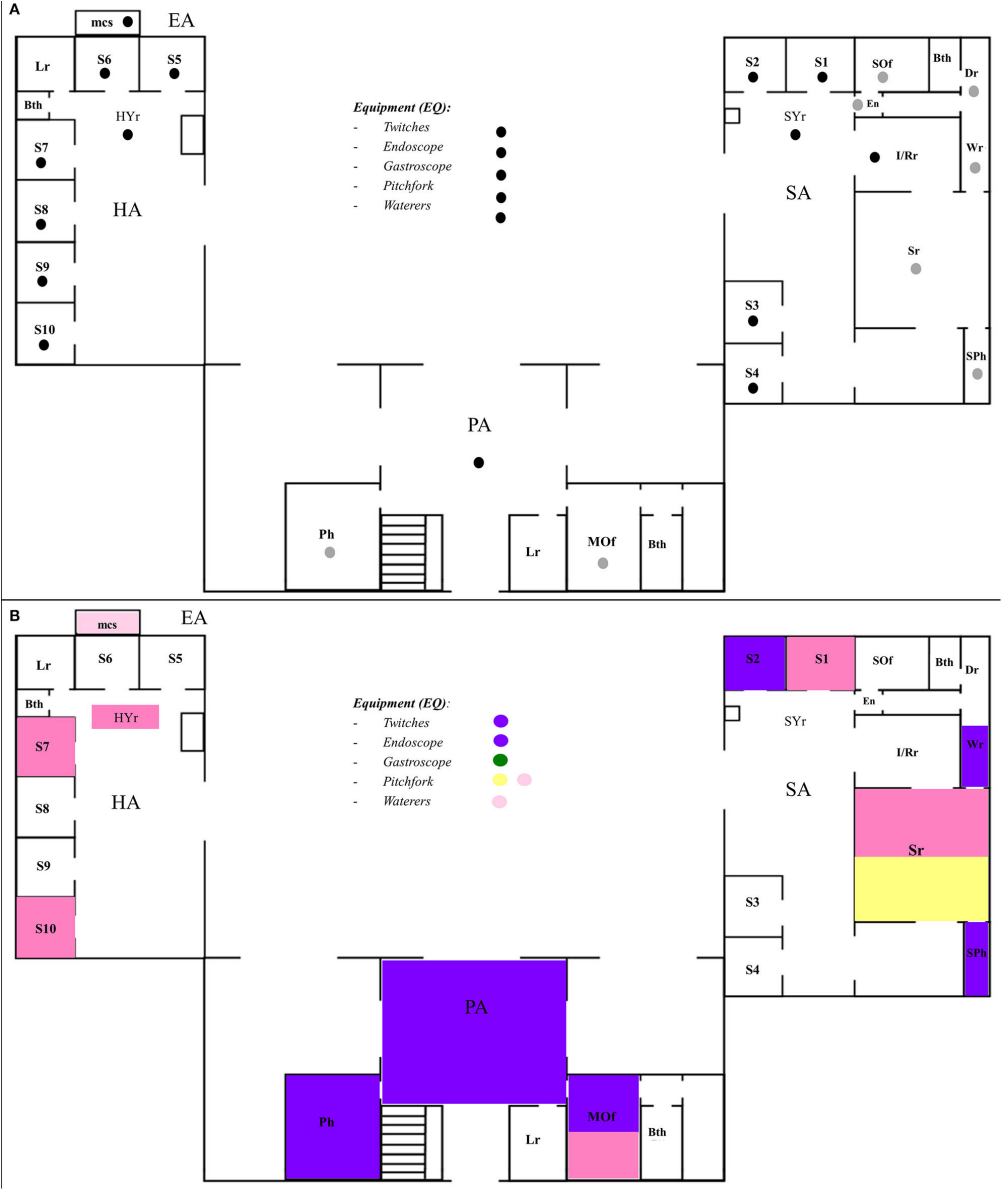
Schematic diagram of the equine veterinary hospital. **(A)** Black circles show the animal contact surfaces, whereas the white circles show the human contact surfaces. **(B)** Locations of the different pulse types of *Salmonella enterica* found in this study (colors matching Figure 2). SA, surgery area; PA, proceeding area; HA, hospitalization area; EA, exterior area; S1–10, stalls; Sr, surgery room; I/Rr, induction/recovery room, SPh, surgery pharmacy; Wr, washing room; SOf, surgery office; En, entrance; Bth, bathroom; Dr, dressing room; MOf, main office; SYr, surgery yard; HYr, hospitalization yard; mcs, manure collection site.

A total of 545 samples were obtained in a longitudinal study conducted from July 2015 to June 2016. With the corresponding consent from the Chief Director, we collected both environmental (*n* = 61, for details see Table 1) samples and patient fecal samples, from one to nine, depending on hospitalized horses at a given time (20, 21). Samples were conducted during the afternoon on the last Friday of every month. The hospital was divided into four areas: surgical area (SA), proceeding area (PA), hospitalization area (HA), exterior area (EA), and a fifth category for equipment (EQ), similarly as described by Alinovi et al. (18) (Figure 1A). In addition, the surfaces sampled were classified into animal contact surfaces (direct contact of animals and humans) (*n* = 396) and human contact surfaces (direct contact of humans, but out of reach of animals) (*n* = 96), as previously described Alinovi et al. (20) (Figure 1A). The samples were obtained using a sterile gauze soaked in 90 ml of peptone water (Becton-Dickinson™, Franklin Lakes, NJ) and rubbed on the surface for 5 min. For patient samples, approximately 100 g of manure was collected and transferred into a sterile recipient. To avoid interference with the normal activities of the EVH, only one sample per hospitalized patient was collected on each sampling day. All the samples were maintained at 4°C during sampling and immediately transferred to the laboratory at Universidad Andres Bello (Santiago, Chile) for further analysis.

**Table 1:** Results of *Salmonella* spp. on samples collected in the equine veterinary hospital during the study.

**Sample origin**	**No. of samples**	**No. positive samples**	**% positive samples**
Animal feces	53	1	1.88
**Environmental/surgery (SA)**^**a**^			
Stalls (1–4)	48	1	2.08
Surgery room floor	12	2	16.67
Bed	12	0	0
Pharmacy	12	1	8.33
Washing room	12	1	8.33
Dressing room	12	0	0
Personal entrance	12	0	0
Office	12	0	0
Induction/recovery room	12	0	0
Area Floor	12	0	0
**Environmental/hospitalization (HA)**^**a**^			
Stalls (5–10)	72	3	4.17
Floor	12	1	8.33
**Environmental/proceeding (PA)**^**a**^			
Pharmacy	12	1	8.33
Floor	12	1	8.33
Main office	12	2^b^	16.67
**Environmental/equipment (EQ)**^**a**^			
Twitches (3×)	36	1	2.78
Endoscope	12	1	8.33
Gastroscope	12	1	8.33
Pitchforks (2×)	24	2	8.33
Waterers (1×)	120	1	0.83
**Environmental/exterior (EA)**^**a**^			
Manure collection site	12	1	8.33
Total	545	21	3.85

a *Environmental samples were classified according to how the hospital was divided into four main areas, plus equipment (see Figure 1 and Materials and Methods)*.

b *Two different isolates were obtained from one sample taken on September 2015*.

**Figure 2:**
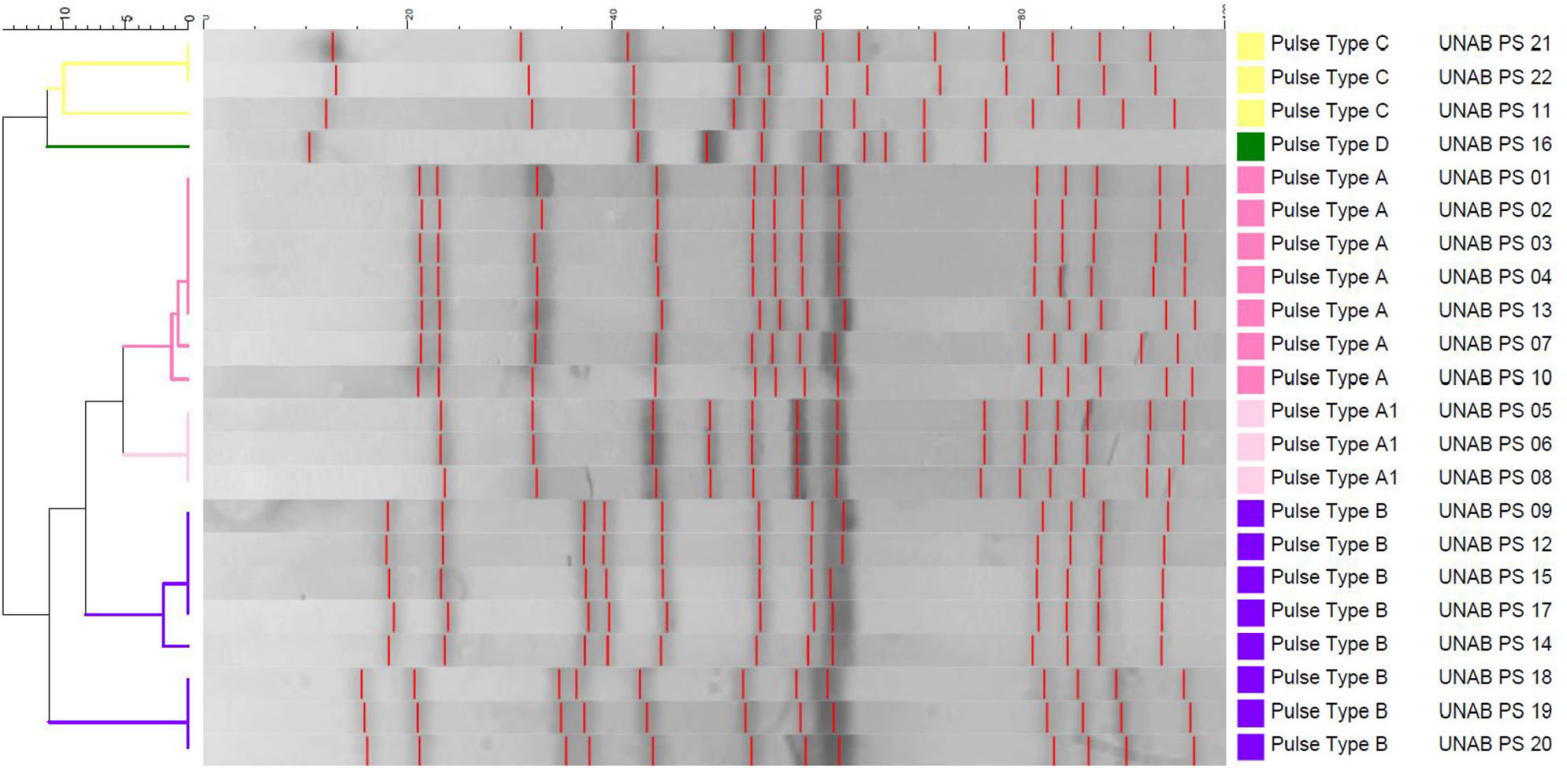
Dendrogram representation of *Salmonella enterica* isolates clustered using unweighted pair group method with arithmetic mean (UPGMA) method. Five pulse types of *Salmonella* were identified at the right; colors have been assigned for each pulse type (A–D), matching Figure 1B.

### Bacterial Culture and Molecular Identification

*Salmonella* isolation was conducted as previously described (22). In brief, all samples were cultured in peptone water at 37°C overnight, and 100 μl and 1 ml were transferred into Rappaport–Vassiliadis (RV) (BD, Franklin Lakes, NJ) supplemented with novobiocin (20 mg/ml) and 100 μl of Tetrathionate (TT) (BD, Franklin Lakes, NJ) supplemented with iodine, respectively, and incubated at 42°C overnight. Finally, 100 μl of aliquot of each selective broth was streaked into an XLT-4 agar plate (BD, Franklin Lakes, NJ) and incubated at 37°C overnight. Four colonies of each agar plate were selected and transferred into Tryptic Soy Agar (TSA) (BD, Franklin Lakes, NJ). All presumed colonies of *Salmonella* spp. were confirmed by *invA*-PCR. Primers and PCR conditions used in this study have been previously described (23). Confirmed colonies were grown overnight in Trypticase Soy Broth (TSB) (BD, Franklin Lakes, NJ) and then immersed in a 20% solution of glycerol (Winkler, Santiago, Chile) and stored at −80°C.

### Determination of Antimicrobial Susceptibility

The disk diffusion method of Kirby–Bauer was used to determine antimicrobial susceptibility (24). PCR-confirmed colonies were suspended in 5 ml of Mueller–Hinton (MH) broth (BD, Franklin Lakes, NJ) and incubated at 37°C overnight. Cultures were adjusted to MacFarland 0.5 (bioMérieux, France) (equivalent to 1.5 × 10^8^ CFU/mL) and streaked on MH agar. An OXOID™ (Hampshire, UK) sensitivity disk dispenser was used, along with the antimicrobial disks, detailed as follows: amikacin (AMK; 30 μg), amoxicillin/clavulanate (AMC; 30 μg), ampicillin (AMP; 10 μg), cefoxitin (FOX; 30 μg), ceftriaxone (CTR; 30 μg), ciprofloxacin (CIP; 5 μg), chloramphenicol (CHL; 30 μg), streptomycin (STR; 300 μg), gentamicin (GEN; 10 μg), kanamycin (KAN; 30 μg), trimethoprim/sulfamethoxazole (SXT; 23.75 μg), and tetracycline (TET; 30 μg). The agar plates were incubated at 37°C overnight. *Escherichia coli* American Type Culture Collection (ATCC) 25922 was used as control. Interpretations were made based on the guidelines of Clinical Laboratory Standard Institute (25). The samples were classified according to Magiorakos's criteria as MDR when resistant to at least one agent in three or more antimicrobial classes (26).

### Molecular Characterization of *Salmonella* Serotype

A previously described molecular method for serotype prediction was used (27, 28). Briefly, DNA extraction of the isolates was conducted using the DNeasy Blood and Tissue kit (QIAGEN, Hilden, Germany). The molecular scheme included an initial multiplex PCR, conducted to identify the serogroup of each isolate, followed by PCR-sequencing approaches to determine H1 and H2 antigens (27, 28). PCR products were sent to MACROGEN™ (Korea) for Sanger sequencing. Consensus sequences were obtained using CAP3 Sequence Assembly Program (http://doua.prabi.fr/software/cap3); the complementary reverse was obtained by using Bioinformatics.org. The results were analyzed using basic local alignment tool (BLAST) on the National Center for Biotechnology Information (NCBI).

### Molecular Typing

Molecular typing of the isolates was conducted by pulsed-field gel electrophoresis (PFGE), using the CDC PulseNet standard protocol (29). For this, overnight cultures in brain hearth infusion broth (BHI, BD, Germany) were embedded in 1% of SeaKem® Gold Agarose (Lonza, Rockland, ME, USA). Upon lysis and washing, the plugs were digested with *Xba*I (Thermo Fisher Scientific Inc., Waltham, MA). The CHEF-DR® III System (Bio-Rad Laboratories, Hercules, CA) was used for the electrophoresis for 20 h. A standard, *Salmonella* Braenderup digested with *Xba*I was used. BioNumerics v 7.5 (Applied Maths, Sint-Martens-Latem, Belgium) (30) was used to analyze the PFGE images using unweighted pair group method with arithmetic mean (UPGMA) and the Dice correlation coefficient. PFGE was conducted at the Microbiology Unit of the Clinical Laboratory Services of Red Salud UC-CHRISTUS, Catholic University. The results were analyzed using Tenover guidelines as previously described (31).

## Results

### *Salmonella* spp. Were Obtained Mostly From Environmental Samples in Human Contact Surfaces

A total of 545 samples (environmental, *n* = 492; patient, *n* = 53) were analyzed. Among these, 21 samples (3.85%) yielded positive for *Salmonella*, which were confirmed by *invA*-PCR (Table 1 and Supplementary Figure 1). In 3/21 (14.2%) samples, *Salmonella* isolates were obtained from TT enrichments; in 10/21 (47.6%), *Salmonella* isolates were obtained from RV enrichments; and in the remaining 10/21 (47.6%) samples, *Salmonella* isolates were obtained from both enrichments conducted. On positive samples, one isolate was selected, except for one sample, in which two different colonies were obtained; therefore, a total of 22 *Salmonella* colonies were further characterized. From the 22 isolates, 1/22 (4.5%) was obtained from a sick Chilean rodeo patient, which died of peritonitis after colic surgery (no positive foreign patients were found), and the other 21 (21/23; 95.4%) were obtained from 20 environmental samples (i.e., stalls, surgery room floor, surgical pharmacy, washing room, hospitalization area floor, main office, pitchforks, endoscope, gastroscope, twitches, waterers, manure collection site, proceeding area floor, and pharmacy) (Table 1). Regarding the type of contact surface, 13/396 (3.28%) isolates were obtained from animal contact surfaces and 8/96 (9.38%) from human contact surfaces (Table 2). About the dates of isolation, two peaks were seen during the months of September 2015 and May 2016, where 9/22 and 8/22 isolates of *Salmonella* spp. were obtained, respectively (Supplementary Figure 1). A few isolates were also obtained during October 2015 (*n* = 1), December 2015 (*n* = 1), April 2016 (*n* = 1), and May 2016 (*n* = 2) (Table 2, Supplementary Figure 1).

**Table 2:** Characteristics, serotypes, PFGE patterns, and antimicrobial resistance of *Salmonella* isolates.

**Isolate ID (UAB)^**a**^**	**Isolation date**	**Source^**b**^**	**Area^**b**^**	**Serotype**	**PFGE pattern**	**Antibiotic resistance profiles^**c**^**
PS-001	Sept 2015	Stall 1	Surgery^d^	Typhimurium	A	AMP
PS-002	Sept 2015	Stall 7	Hospitalization^d^	Typhimurium	A	AMP
PS-003	Sept 2015	Stall 10	Hospitalization^d^	Typhimurium	A	AMP
PS-004	Sept 2015	Yard	Hospitalization^d^	Typhimurium	A	AMP
PS-005	Sept 2015	Pitchfork	Equipment^d^	Typhimurium	A1	AMP
PS-006	Sept 2015	Manure collection site	Exterior^d^	Typhimurium	A1	AMP
PS-007	Sept 2015	Main office	Proceeding^e^	Typhimurium	A	Pan-susceptible
PS-008	Sept 2015	Waterers	Equipment^d^	Typhimurium	A1	STR
PS-009	Sept 2015	Main office	Proceeding^e^	Typhimurium	B	AMC–AMP–CHL–STR–TET
PS-010	Oct 2015	Stall 10	Hospitalization^d^	Typhimurium	A	AMP
PS-011	Dec 2015	Surgery room floor	Surgery^e^	Infantis	C	AMP
PS-012	Apr 2016	Pharmacy	Proceeding^e^	Typhimurium	B	AMC–AMP–CIP–CHL–STR–GEN–SXT–TET
PS-013	May 2016	Surgery room floor	Surgery^e^	Typhimurium	A	AMC–AMP–CHL–STR–TET
PS-014	May 2016	Twitch	Equipment^d^	Typhimurium	B	AMC–AMP–CTR–CHL–STR–TET
PS-015	May 2016	Endoscope	Equipment^d^	Typhimurium	B	AMC–AMP–CIP–CHL–STR–GEN–SXT–TET
PS-016	May 2016	Gastroscope	Equipment^d^	Typhimurium	D	Pan-susceptible
PS-017	May 2016	Proceeding area floor	Proceeding^d^	Typhimurium	B	AMC–AMP–CHL–STR–TET
PS-018	May 2016	Main office	Proceeding^e^	Typhimurium	B	AMC–AMP–CHL–STR–TET
PS-019	May 2016	Washing room	Surgery^e^	Typhimurium	B	AMC–AMP–CHL–STR–TET
PS-020	May 2016	Pharmacy	Surgery^e^	Typhimurium	B	AMC–AMP–CHL–STR–TET
PS-021	Jun 2016	Pitchfork	Equipment^d^	Infantis	C	AMP
PS-022	Jun 2016	Patient	Surgery^d^	Infantis	C	AMP

*PFGE, pulsed-field gel electrophoresis*.

a *All isolates with pre-fix UAB after Universidad Andres Bello laboratory*.

b *Sources and areas in the hospital where the samples were taken (see Figure 1)*.

c *Amikacin (AMK), amoxicillin/clavulanate (AMC), ampicillin (AMP), cefoxitin (FOX), ceftriaxone (CTR), ciprofloxacin (CIP), chloramphenicol (CHL), streptomycin (STR), gentamicin (GEN), kanamycin (KAN), trimethoprim/sulfamethoxazole (SXT), and tetracycline (TET)*.

d *Animal contact surfaces*.

e *Human contact surfaces*.

### Presence of Multidrug-Resistant *Salmonella* Isolates

Kirby–Bauer tests revealed six antimicrobial resistant profiles (Table 2). From the 22 *Salmonella* isolates, two were pan-susceptible, 10 isolates were resistant to AMP; one isolate was resistant to STR; six isolates were resistant to AMC, AMP, CHL, STR, and TET; one isolate was resistant to AMC, AMP, CTR, CHL, STR, and TET; and two isolates were resistant to AMC, AMP, CIP, CHL, STR, GEN, SXT, and TET. From these, 9/22 (40.1%) were classified as MDR, as these were resistant to one agent in three or more antimicrobial classes (26).

### Predominance of *Salmonella* Serotype Typhimurium

All isolates were tested to predict the serogroup and serotype as described above. The molecular methods showed 19/22 (86.4%) of *Salmonella* isolates to O:4 (B) serogroup and three *Salmonella* isolates 3/22 (13.6%) to O:7 (C1) serogroup. Concerning flagellar antigens, DNA was amplified for both genes, *fli*C and *flj*B, in all *Salmonella* isolates. The BLAST algorithm of the FASTA consensus sequences of the PCR products allowed us to predict the serotype. All isolates belonging to O:4 (B) serogroup (20/22) yielded positive for serotype Typhimurium, whereas the isolates belonging to O:7 (C1) serogroup (2/22) were predicted as Infantis serotype (Table 2).

### Five Different Pulsed-Field Gel Electrophoresis Types of *Salmonella* Were Identified

According to the PFGE, four PFGE patterns were identified in 19 *Salmonella typhimurium* isolates, and one PFGE type was found in three *S*. Infantis isolates. Among *S*. Typhimurium, seven isolates (1, 2, 3, 4, 7, 10, and 13) were indistinguishable from each other and classified as PFGE pattern A. In three isolates (5, 6, and 8), PFGE patterns were also indistinguishable from each other and related to PFGE pattern A, which was therefore classified as A1. All PFGE patterns A and A1 were detected only in the sampling of September 2015. Eight isolates (9, 12, 14, 15, 17, 18, 19, and 20) were indistinguishable from each other and different from all others, classified as PFGE pattern B; these isolates were obtained in samplings of September 2015 and in April and May 2016. One additional PFGE pattern D of isolate 16 was found in *S*. Typhimurium. Isolates 11, 21, and 22 were indistinguishable from each other and different from all others, classified as PFGE pattern C. Importantly, these isolates were classified as *S*. Infantis (Table 2).

## Discussion

This study examined the environmental presence of *Salmonella* in an equine hospital with no history of outbreak or hospital-acquired infections. Here, we identified two serotypes that were widely distributed. The major findings of this study are the following: (i) wide spatial distribution of *Salmonella* in the hospital, mainly in spring and autumn; (ii) MDR *Salmonella* Typhimurium accounted for most of the isolates; and (iii) multiple *Salmonella* PFGE patterns present in human contact surfaces highlight the need of developing biosecurity standard protocols.

### Wide Spatial Distribution of *Salmonella* in the Hospital, Mainly in Spring and Autumn

In this study, we found a considerable presence of *Salmonella* in the EVH environment, compared with the equine's samples. The prevalence of *Salmonella* in equine subclinical shedders (1–2%) tends to increase under stress conditions owing to hospitalization to 9–13% (5, 6, 10). In the environmental samples, positivity was widespread to all sampled areas (including equipment), reaching 4.5%. A previous study conducted at a large animal hospital has shown the presence of *Salmonella* in several areas, accounting for a positivity rate of 3.9% during a post-outbreak period (32). Importantly, in our study, no outbreak or hospital-acquired infections were reported, before and/or during the study.

It has been shown that the peak incidence of salmonellosis in horses occurs in summer and autumn (5, 33), although there are some outbreak reports during spring (7). Here, we obtained *Salmonella* isolates in every season of the year, although the highest number of isolates was obtained during September 2015 and June 2016, spring and winter for the southern hemisphere, respectively (Table 2, Supplementary Figure 2). Our first peak, on September 2015, was an incoming Chilean rodeo patient suffering from severe acute diarrhea, which died within 24 h after being admitted to the EVH. As *Salmonella* was isolated from the stall of that patient, Stall 10 (Figure 1), it may have been introduced to the EVH by this patient, but further investigation is needed, which is beyond the scope of this study. Importantly, these isolates represented a closely related PFGE pattern. Nevertheless, neither official information nor patient history could be collected to explain the second peak, in June 2016. Although it is uncertain about the origin of these isolates, shedding patients present during non-sampling periods could be a common source of dissemination (5). Other possible sources of contaminations, such as other animals (rodents), feed, or even environmental persistent strains (34), are also plausible and have to be considered.

### Multidrug-Resistant *Salmonella* Typhimurium Accounted for Most Isolates

Reported outbreaks of *Salmonella* in EVHs have involved serotypes such as Typhimurium, Newport, Agona, Anatum (12, 35), Infantis (36), Heidelberg (37), and Oranienburg (38). Here, we found that 87% of the isolates were represented by *S*. Typhimurium. This serotype has been commonly isolated from horses, causing severe clinical signs, along with high morbidity and mortality rates (7, 8, 33). In Chile, only one outbreak of *S*. Typhimurium has been reported, which affected weanling foals with a morbidity rate of 87% and mortality rate of 13% (39). Regarding *Salmonella* Infantis, which is less commonly reported compared with *S*. Typhimurium, only three isolates were found. Nonetheless, there is a report of a serious outbreak in 1996, which caused important economic losses and even the closure of the facilities (36).

Antimicrobial resistance profiles, which include resistance to AMP (10 isolates), as the most common profile, followed by the profile AMC–AMP–CRO–CHL–STR–TE (six isolates), include antimicrobials in which resistance has already been described in other salmonellosis outbreaks (38), not only in equine hospitals but also in small animal shelters (13). Notably, we found that almost half of the isolates (*n* = 10) displayed an MDR phenotype, showing resistance to three or more antimicrobial classes (26), which is a major concern for the public health, the personnel at the hospital, and the treatment of hospitalized horses.

### Multiple *Salmonella* Pulsed-Field Gel Electrophoresis Patterns Present in Human Contact Surfaces Highlight the Need of Developing Biosecurity Standards

We found five different PFGE patterns, which were present in all areas of the hospital, including human contact surfaces. Environmental presence of *Salmonella* indicates that personnel without animal contact at all (e.g., secretary) could also be at risk of infection. As pointed before, no information concerning hospital-acquired infections was reported during our study, neither from incoming patients nor from veterinary staff. In the environment, *Salmonella* could put into high risk the incoming susceptible patients, as young horses or immunocompromised individuals (33). This leads us to think that it may be a potential risk of an outbreak. There has been reports of $755,000 USD of estimated cost to control salmonellosis outbreaks in a large animal teaching hospital in Virginia (USA) (12), which lead us to the conclusion that biosecurity standard protocols must be implemented to prevent any undesirable event (17). There are many guidelines of biosecurity protocols (e.g., rubber boots, hand washing, and foot bath) (21, 40, 41) and also published articles in which salmonellosis outbreaks have been controlled (7, 12, 16, 18, 19). Although the implementation of biosecurity protocols is quite expensive, it is much less than controlling an outbreak itself, especially considering the fact that the EVH located at a thoroughbred racetrack, harbors nearly 1,500 horses together with hospital personnel (17).

## Conclusions

This study has revealed the importance of implementing mitigation strategies and biosecurity protocols to control MDR *Salmonella* to ensure the safety of patients and hospital personnel. Also, this could set an example for other veterinary facilities to establish or recheck their functioning biosecurity protocols, especially in developing countries.

## Data Availability Statement

The datasets generated for this study are available on request to the corresponding author.

## Ethics Statement

The animal study was reviewed and approved by The University Andres Bello Bioethics Committee, Santiago, Chile. Written informed consent for participation was not obtained from the owners because fresh fecal samples were obtained from the floor.

## Author Contributions

PS-O designed the study, conducted the experiments, and wrote the manuscript. AM-S wrote the manuscript, analyzed data, and designed the study. DR and RT conducted the experiments. RR-N critically reviewed the manuscript. AA, GG-R, and CH-W analyzed the data. PG conducted the experiments. All authors contributed to the article and approved the submitted version.

## Conflict of Interest

The authors declare that the research was conducted in the absence of any commercial or financial relationships that could be construed as a potential conflict of interest.
