# Supplementary figures and images for: Widespread Environmental Presence of Multidrug-Resistant Salmonella in an Equine Veterinary Hospital That Received Local and International Horses
Source: Front Vet Sci. 2020 Jul 10;7:346. doi: 10.3389/fvets.2020.00346 (PMC7366320; doi:10.3389/fvets.2020.00346)

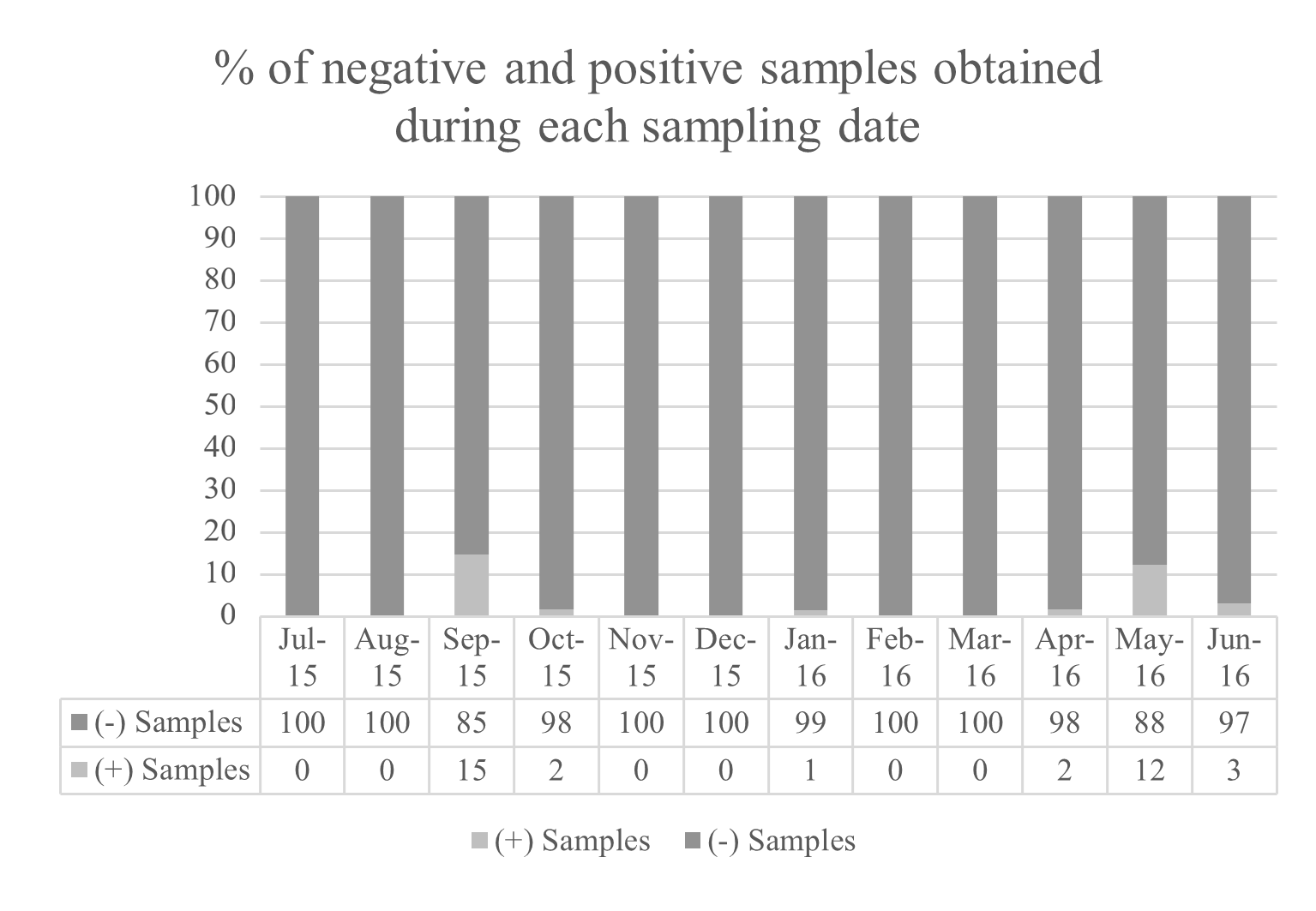

Supplement: Supplementary file 1 [file Image_1.TIF]

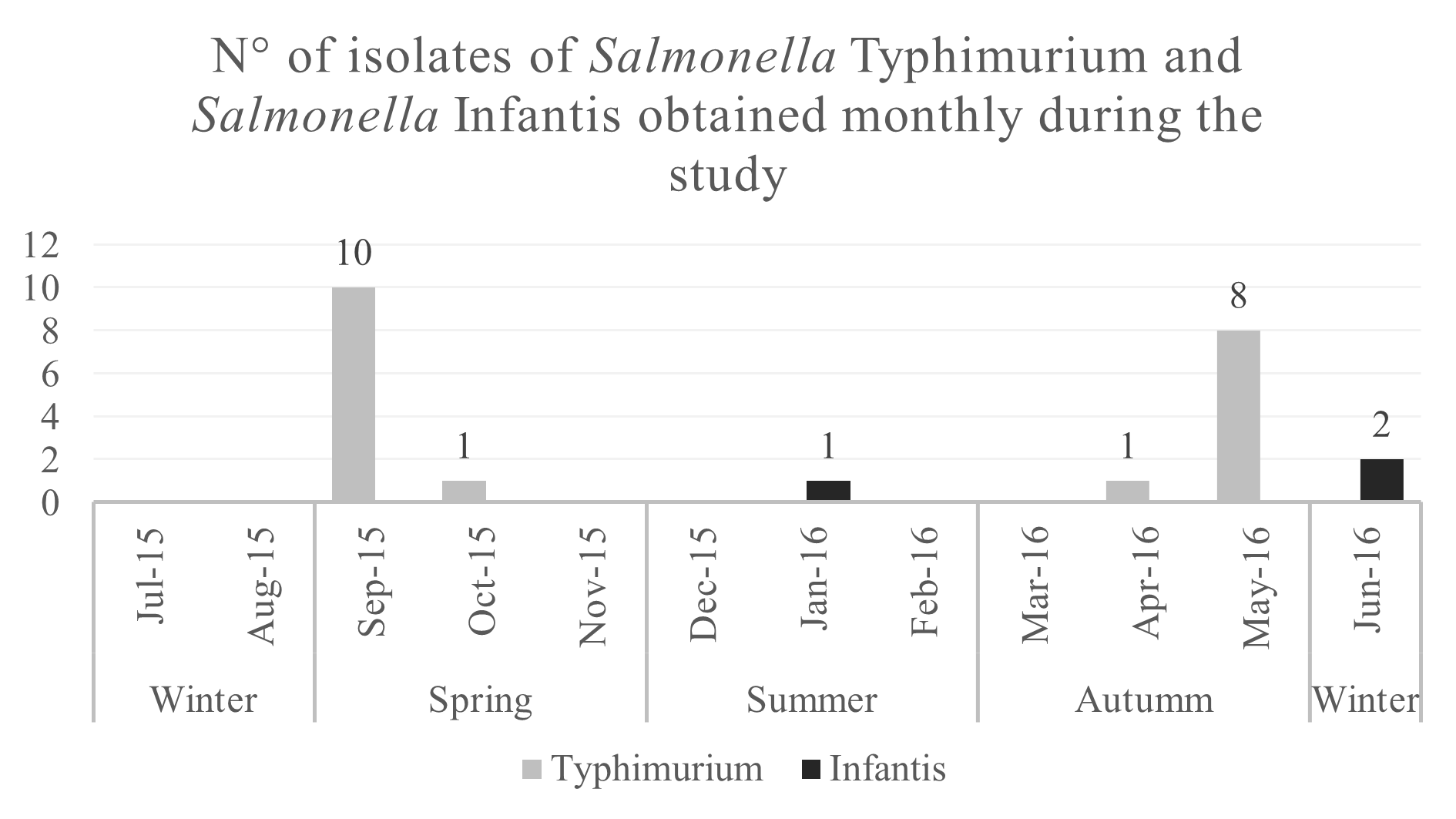

Supplement: Supplementary file 2 [file Image_2.TIF]
